# Supplementary material for: Use of Fourier Series in X-ray Diffraction (XRD) Analysis and Fourier-Transform Infrared Spectroscopy (FTIR) for Estimation of Crystallinity in Cellulose from Different Sources
Source: Polymers (Basel). 2022 Nov 29;14(23):5199. doi: 10.3390/polym14235199 (PMC9736003; doi:10.3390/polym14235199)
Supplement: Supplementary file 1 [file polymers-14-05199-s001.zip › polymers-2055275-supplementary.pdf]

Supplementary Mtaerials

Table S1. XRD data of amorphous cellulose.

Col(A)	Col(B)	Col(C)	Col(D)	Col(E)	Col(F)
**Diffraction Angle 2θ (°)**	**Diffracted Intensity**	**Diffraction Angle 2θ (°)**	**Diffracted Intensity (a.u.)**	**Diffracted Intensity (a.u.)**	**Diffracted Intensity (a.u.)**
			**Zero Background**	**Background Substraction**	**Cellulose powder from banana rachis (P_BR_BM_6.5 h)**
				**Col(B)-Col(D)**	**2·Col(E)/(1+(cos(Radians(Col(A)))²**
10.01304	7149	10.01304	3673	3476	3529.34887
10.0393	7172	10.0393	3776	3396	3448.39617
10.06556	7248	10.06556	3662	3586	3641.61893
10.09182	7212	10.09182	3684	3528	3583.00669
10.11808	7114	10.11808	3694	3420	3473.6021
10.14434	6999	10.14434	3648	3351	3403.79503
10.1706	7174	10.1706	3658	3516	3571.68325
10.19686	7278	10.19686	3641	3637	3694.89891
10.22312	7287	10.22312	3634	3653	3711.4551
10.24938	7387	10.24938	3606	3781	3841.81621
10.27564	7150	10.27564	3619	3531	3588.08797
10.3019	7236	10.3019	3479	3757	3818.05436
10.32816	7277	10.32816	3608	3669	3728.93027
10.35442	7261	10.35442	3518	3743	3804.45196
10.38069	7135	10.38069	3491	3644	3704.13207
10.40695	7307	10.40695	3616	3691	3752.21786
10.43321	6991	10.43321	3538	3453	3510.56142
10.45947	7229	10.45947	3607	3622	3682.68463
10.48573	7355	10.48573	3404	3951	4017.53148
10.51199	7183	10.51199	3486	3697	3759.56826
10.53825	7431	10.53825	3532	3899	3965.31884
10.56451	7280	10.56451	3429	3851	3916.83108
10.59077	7091	10.59077	3518	3573	3634.38452
10.61703	7271	10.61703	3434	3837	3903.24921
10.64329	7175	10.64329	3481	3694	3758.09786
10.66955	7184	10.66955	3458	3726	3790.97437
10.69581	7051	10.69581	3444	3607	3670.211
10.72207	7148	10.72207	3424	3724	3789.58405
10.74833	7245	10.74833	3445	3800	3867.2526
10.77459	7204	10.77459	3376	3828	3896.08151
10.80085	7269	10.80085	3226	4043	4115.25827
10.82712	7374	10.82712	3373	4001	4072.8578
10.85338	7259	10.85338	3318	3941	4012.12597
10.87964	7337	10.87964	3417	3920	3991.09175
10.9059	7222	10.9059	3333	3889	3959.87245
10.93216	7243	10.93216	3335	3908	3979.56412
10.95842	7310	10.95842	3376	3934	4006.38882
10.98468	7211	10.98468	3296	3915	3987.38695
11.01094	7378	11.01094	3396	3982	4055.98033
11.0372	7168	11.0372	3284	3884	3956.5063
11.06346	7198	11.06346	3401	3797	3868.22193
11.08972	7225	11.08972	3341	3884	3957.20221
11.11598	7232	11.11598	3350	3882	3955.51356
11.14224	7250	11.14224	3297	3953	4028.21437
11.1685	7426	11.1685	3367	4059	4136.59798

11.19476	7174	11.19476	3260	3914	3989.18041
11.22102	7273	11.22102	3332	3941	4017.05681
11.24728	7129	11.24728	3220	3909	3984.79498
11.27354	7245	11.27354	3159	4086	4165.59971
11.29981	7407	11.29981	3127	4280	4363.7704
11.32607	7321	11.32607	3215	4106	4186.74112
11.35233	7234	11.35233	3216	4018	4097.37982
11.37859	7428	11.37859	3230	4198	4281.32252
11.40485	7271	11.40485	3264	4007	4086.90141
11.43111	7299	11.43111	3230	4069	4150.51422
11.45737	7390	11.45737	3166	4224	4309.01109
11.48363	7404	11.48363	3221	4183	4267.57481
11.50989	7286	11.50989	3189	4097	4180.21778
11.53615	7224	11.53615	3065	4159	4243.86557
11.56241	7204	11.56241	3166	4038	4120.77457
11.58867	7349	11.58867	3045	4304	4392.63117
11.61493	7088	11.61493	3155	3933	4014.36119
11.64119	7339	11.64119	3183	4156	4242.36615
11.66745	7248	11.66745	2994	4254	4342.80468
11.69371	7326	11.69371	3072	4254	4343.20758
11.71997	7370	11.71997	3177	4193	4281.32644
11.74623	7362	11.74623	3139	4223	4312.36022
11.7725	7426	11.7725	3135	4291	4382.20835
11.79876	7446	11.79876	3008	4438	4532.75716
11.82502	7270	11.82502	3034	4236	4326.85003
11.85128	7383	11.85128	3092	4291	4383.44166
11.87754	7326	11.87754	3031	4295	4387.9412
11.9038	7484	11.9038	3030	4454	4550.81151
11.93006	7413	11.93006	3127	4286	4379.57426
11.95632	7490	11.95632	3162	4328	4422.91062
11.98258	7255	11.98258	3013	4242	4335.43667
12.00884	7236	12.00884	3013	4223	4316.42922
12.0351	7249	12.0351	2970	4279	4374.08561
12.06136	7292	12.06136	2978	4314	4410.28517
12.08762	7337	12.08762	3014	4323	4419.90968
12.11388	7372	12.11388	2984	4388	4486.79776
12.14014	7258	12.14014	2907	4351	4449.39298
12.1664	7240	12.1664	2949	4291	4388.45947
12.19266	7482	12.19266	2969	4513	4615.94786
12.21893	7308	12.21893	2916	4392	4492.62289
12.24519	7338	12.24519	3001	4337	4436.79353
12.27145	7144	12.27145	2958	4186	4282.73568
12.29771	7312	12.29771	2923	4389	4490.86468
12.32397	7416	12.32397	3006	4410	4512.79294
12.35023	7432	12.35023	2964	4468	4572.59251
12.37649	7497	12.37649	2882	4615	4723.49705
12.40275	7413	12.40275	2882	4531	4637.97819
12.42901	7271	12.42901	2949	4322	4424.4795
12.45527	7275	12.45527	2870	4405	4509.89273
12.48153	7304	12.48153	2887	4417	4522.62586
12.50779	7431	12.50779	2865	4566	4675.65245
12.53405	7299	12.53405	2735	4564	4674.0687
12.56031	7385	12.56031	2871	4514	4623.32305
12.58657	7319	12.58657	2841	4478	4586.90868
12.61283	7401	12.61283	2768	4633	4746.15276
12.63909	7457	12.63909	2860	4597	4709.74521

12.66535	7302	12.66535	2819	4483	4593.41023
12.69162	7301	12.69162	2781	4520	4631.78726
12.71788	7306	12.71788	2780	4526	4638.40304
12.74414	7343	12.74414	2756	4587	4701.39266
12.7704	7240	12.7704	2681	4559	4673.16718
12.79666	7306	12.79666	2859	4447	4558.82462
12.82292	7341	12.82292	2771	4570	4685.39354
12.84918	7474	12.84918	2811	4663	4781.22848
12.87544	7300	12.87544	2764	4536	4651.48286
12.9017	7289	12.9017	2779	4510	4625.2936
12.92796	7355	12.92796	2797	4558	4674.99939
12.95422	7366	12.95422	2711	4655	4774.97922
12.98048	7542	12.98048	2630	4912	5039.12127
13.00674	7287	13.00674	2771	4516	4633.35019
13.033	7359	13.033	2777	4582	4701.55051
13.05926	7511	13.05926	2802	4709	4832.36388
13.08552	7352	13.08552	2739	4613	4734.33952
13.11178	7275	13.11178	2701	4574	4694.80113
13.13804	7193	13.13804	2748	4445	4562.86888
13.16431	7446	13.16431	2688	4758	4884.67793
13.19057	7295	13.19057	2761	4534	4655.20031
13.21683	7324	13.21683	2626	4698	4824.08909
13.24309	7461	13.24309	2666	4795	4924.20877
13.26935	7321	13.26935	2663	4658	4784.01967
13.29561	7505	13.29561	2587	4918	5051.58556
13.32187	7346	13.32187	2631	4715	4843.58237
13.34813	7424	13.34813	2729	4695	4823.54664
13.37439	7411	13.37439	2656	4755	4885.70667
13.40065	7400	13.40065	2678	4722	4852.31426
13.42691	7382	13.42691	2586	4796	4928.88029
13.45317	7399	13.45317	2603	4796	4929.40519
13.47943	7371	13.47943	2594	4777	4910.40056
13.50569	7355	13.50569	2614	4741	4873.91621
13.53195	7397	13.53195	2654	4743	4876.49453
13.55821	7418	13.55821	2547	4871	5008.63458
13.58447	7464	13.58447	2717	4747	4881.65563
13.61074	7480	13.61074	2573	4907	5046.73783
13.637	7456	13.637	2571	4885	5024.65354
13.66326	7328	13.66326	2554	4774	4911.01119
13.68952	7352	13.68952	2500	4852	4991.79044
13.71578	7467	13.71578	2603	4864	5004.67928
13.74204	7469	13.74204	2548	4921	5063.87843
13.7683	7462	13.7683	2562	4900	5042.81801
13.79456	7415	13.79456	2563	4852	4993.96397
13.82082	7410	13.82082	2566	4844	4986.27507
13.84708	7478	13.84708	2498	4980	5126.83116
13.87334	7382	13.87334	2518	4864	5007.96058
13.8996	7514	13.8996	2445	5069	5219.60187
13.92586	7414	13.92586	2517	4897	5043.04717
13.95212	7284	13.95212	2521	4763	4905.59212
13.97838	7299	13.97838	2665	4634	4773.2579
14.00464	7272	14.00464	2526	4746	4889.16516
14.0309	7240	14.0309	2535	4705	4847.46627
14.05716	7412	14.05716	2458	4954	5104.57338
14.08343	7346	14.08343	2417	4929	5079.3792
14.10969	7298	14.10969	2485	4813	4960.39359

14.13595	7452	14.13595	2504	4948	5100.0979
14.16221	7380	14.16221	2413	4967	5120.2553
14.18847	7392	14.18847	2393	4999	5153.82082
14.21473	7548	14.21473	2495	5053	5210.07875
14.24099	7277	14.24099	2369	4908	5061.14107
14.26725	7470	14.26725	2468	5002	5158.65593
14.29351	7386	14.29351	2439	4947	5102.50996
14.31977	7456	14.31977	2363	5093	5253.6942
14.34603	7429	14.34603	2426	5003	5161.43983
14.37229	7323	14.37229	2409	4914	5070.19728
14.39855	7459	14.39855	2381	5078	5240.00655
14.42481	7569	14.42481	2344	5225	5392.31116
14.45107	7471	14.45107	2436	5035	5196.82065
14.47733	7465	14.47733	2395	5070	5233.54431
14.50359	7548	14.50359	2439	5109	5274.40688
14.52985	7363	14.52985	2379	4984	5145.95079
14.55612	7520	14.55612	2281	5239	5409.85906
14.58238	7478	14.58238	2361	5117	5284.48919
14.60864	7453	14.60864	2315	5138	5306.78911
14.6349	7285	14.6349	2445	4840	4999.57758
14.66116	7445	14.66116	2403	5042	5208.84099
14.68742	7404	14.68742	2298	5106	5275.57093
14.71368	7396	14.71368	2431	4965	5130.48466
14.73994	7560	14.73994	2357	5203	5377.04337
14.7662	7586	14.7662	2377	5209	5383.87207
14.79246	7476	14.79246	2512	4964	5131.24671
14.81872	7454	14.81872	2389	5065	5236.26249
14.84498	7386	14.84498	2388	4998	5167.60293
14.87124	7547	14.87124	2336	5211	5388.46381
14.8975	7482	14.8975	2354	5128	5303.26115
14.92376	7581	14.92376	2343	5238	5417.65917
14.95002	7404	14.95002	2390	5014	5186.58849
14.97628	7560	14.97628	2394	5166	5344.45258
15.00254	7509	15.00254	2287	5222	5403.0271
15.02881	7544	15.02881	2272	5272	5455.40779
15.05507	7655	15.05507	2285	5370	5557.47773
15.08133	7556	15.08133	2289	5267	5451.53092
15.10759	7439	15.10759	2315	5124	5304.15352
15.13385	7453	15.13385	2260	5193	5376.22181
15.16011	7455	15.16011	2285	5170	5353.05096
15.18637	7547	15.18637	2278	5269	5456.21028
15.21263	7640	15.21263	2257	5383	5574.93021
15.23889	7560	15.23889	2230	5330	5520.70456
15.26515	7593	15.26515	2231	5362	5554.51875
15.29141	7442	15.29141	2236	5206	5393.56863
15.31767	7587	15.31767	2205	5382	5576.58395
15.34393	7558	15.34393	2264	5294	5486.06666
15.37019	7515	15.37019	2177	5338	5532.33403
15.39645	7524	15.39645	2283	5241	5432.46269
15.42271	7431	15.42271	2261	5170	5359.52119
15.44897	7617	15.44897	2199	5418	5617.29709
15.47524	7632	15.47524	2172	5460	5661.53331
15.5015	7500	15.5015	2232	5268	5463.11458
15.52776	7601	15.52776	2197	5404	5604.83833
15.55402	7497	15.55402	2237	5260	5456.15611
15.58028	7615	15.58028	2111	5504	5709.95717

15.60654	7522	15.60654	2201	5321	5520.78905
15.6328	7550	15.6328	2138	5412	5615.89836
15.65906	7579	15.65906	2072	5507	5715.18341
15.68532	7736	15.68532	2197	5539	5749.10437
15.71158	7689	15.71158	2157	5532	5742.55044
15.73784	7700	15.73784	2125	5575	5787.90542
15.7641	7485	15.7641	2217	5268	5469.86131
15.79036	7589	15.79036	2164	5425	5633.57879
15.81662	7470	15.81662	2167	5303	5507.57508
15.84288	7613	15.84288	2099	5514	5727.43037
15.86914	7476	15.86914	2204	5272	5476.74855
15.8954	7649	15.8954	2090	5559	5775.61859
15.92166	7545	15.92166	2122	5423	5635.0264
15.94793	7672	15.94793	2078	5594	5813.44301
15.97419	7749	15.97419	2103	5646	5868.22186
16.00045	7643	16.00045	2225	5418	5631.95833
16.02671	7637	16.02671	2105	5532	5751.18677
16.05297	7613	16.05297	2159	5454	5670.81383
16.07923	7708	16.07923	2046	5662	5887.82867
16.10549	7678	16.10549	2245	5433	5650.41225
16.13175	7737	16.13175	2121	5616	5841.47801
16.15801	7606	16.15801	2085	5521	5743.39518
16.18427	7682	16.18427	2100	5582	5807.59304
16.21053	7660	16.21053	2029	5631	5859.32181
16.23679	7738	16.23679	2131	5607	5835.09521
16.26305	7582	16.26305	2122	5460	5682.84338
16.28931	7613	16.28931	2014	5599	5828.2645
16.31557	7699	16.31557	2055	5644	5875.86242
16.34183	7754	16.34183	2130	5624	5855.79469
16.36809	7463	16.36809	2026	5437	5661.81748
16.39435	7751	16.39435	2033	5718	5955.20578
16.42062	7806	16.42062	2039	5767	6007.01546
16.44688	7861	16.44688	2060	5801	6043.21337
16.47314	7687	16.47314	2029	5658	5895.00743
16.4994	7685	16.4994	1997	5688	5927.03429
16.52566	7803	16.52566	2109	5694	5934.05873
16.55192	7784	16.55192	2045	5739	5981.73562
16.57818	7799	16.57818	1891	5908	6158.68761
16.60444	7861	16.60444	2016	5845	6093.81115
16.6307	7686	16.6307	1971	5715	5959.0576
16.65696	7714	16.65696	1987	5727	5972.35332
16.68322	7818	16.68322	1994	5824	6074.3068
16.70948	7607	16.70948	1975	5632	5874.82775
16.73574	7874	16.73574	2007	5867	6120.76634
16.762	7730	16.762	1987	5743	5992.19361
16.78826	7804	16.78826	1946	5858	6112.99138
16.81452	7657	16.81452	1980	5677	5924.89683
16.84078	7719	16.84078	2022	5697	5946.55838
16.86705	7729	16.86705	1975	5754	6006.85268
16.89331	7738	16.89331	1957	5781	6035.84162
16.91957	7782	16.91957	1939	5843	6101.38714
16.94583	7925	16.94583	1997	5928	6190.97154
16.97209	7991	16.97209	1949	6042	6310.87149
16.99835	7862	16.99835	2026	5836	6096.51981
17.02461	7924	17.02461	1977	5947	6213.30713
17.05087	7875	17.05087	1990	5885	6149.35569

17.07713	7944	17.07713	1935	6009	6279.76947
17.10339	7971	17.10339	1810	6161	6439.48511
17.12965	7898	17.12965	1851	6047	6321.18393
17.15591	7803	17.15591	1991	5812	6076.34851
17.18217	7876	17.18217	1951	5925	6195.32539
17.20843	7952	17.20843	1963	5989	6263.09305
17.23469	7904	17.23469	1928	5976	6250.3453
17.26095	7995	17.26095	1915	6080	6359.98305
17.28721	7926	17.28721	1890	6036	6314.81532
17.31347	7968	17.31347	1912	6056	6336.60186
17.33974	8049	17.33974	1856	6193	6480.83331
17.366	7947	17.366	1938	6009	6289.14024
17.39226	7990	17.39226	1850	6140	6427.12633
17.41852	8033	17.41852	1870	6163	6452.08545
17.44478	8174	17.44478	1819	6355	6654.00405
17.47104	8105	17.47104	1835	6270	6565.90655
17.4973	8087	17.4973	1871	6216	6510.25349
17.52356	7992	17.52356	1851	6141	6432.58915
17.54982	8019	17.54982	1930	6089	6378.99997
17.57608	7987	17.57608	1918	6069	6358.92583
17.60234	8060	17.60234	1836	6224	6522.23269
17.6286	8268	17.6286	1803	6465	6775.71925
17.65486	8144	17.65486	1835	6309	6613.13908
17.68112	8041	17.68112	1865	6176	6474.62707
17.70738	8163	17.70738	1838	6325	6631.75432
17.73364	8161	17.73364	1868	6293	6599.12182
17.7599	8259	17.7599	1779	6480	6796.16667
17.78616	8101	17.78616	1792	6309	6617.74807
17.81243	8047	17.81243	1784	6263	6570.41631
17.83869	8126	17.83869	1891	6235	6541.95864
17.86495	8212	17.86495	1827	6385	6700.28358
17.89121	8301	17.89121	1812	6489	6810.37599
17.91747	8127	17.91747	1786	6341	6655.98274
17.94373	8323	17.94373	1810	6513	6837.4902
17.96999	8184	17.96999	1870	6314	6629.51119
17.99625	8362	17.99625	1777	6585	6915.0303
18.02251	8194	18.02251	1807	6387	6708.05613
18.04877	8258	18.04877	1753	6505	6832.95597
18.07503	8369	18.07503	1756	6613	6947.38682
18.10129	8202	18.10129	1762	6440	6766.60067
18.12755	8159	18.12755	1737	6422	6748.6482
18.15381	8311	18.15381	1766	6545	6878.88476
18.18007	8266	18.18007	1750	6516	6849.3828
18.20633	8395	18.20633	1736	6659	7000.69964
18.23259	8371	18.23259	1709	6662	7004.85599
18.25886	8487	18.25886	1717	6770	7119.43436
18.28512	8279	18.28512	1752	6527	6864.87699
18.31138	8182	18.31138	1722	6460	6795.38513
18.33764	8379	18.33764	1733	6646	6992.04784
18.3639	8466	18.3639	1715	6751	7103.53861
18.39016	8445	18.39016	1738	6707	7058.25936
18.41642	8685	18.41642	1654	7031	7400.29715
18.44268	8315	18.44268	1722	6593	6940.29575
18.46894	8324	18.46894	1719	6605	6953.93537
18.4952	8575	18.4952	1662	6913	7279.26278
18.52146	8418	18.52146	1602	6816	7178.16639

18.54772	8571	18.54772	1622	6949	7319.2981
18.57398	8472	18.57398	1691	6781	7143.38633
18.60024	8627	18.60024	1702	6925	7296.1462
18.6265	8597	18.6265	1665	6932	7304.58834
18.65276	8674	18.65276	1639	7035	7414.20895
18.67902	8528	18.67902	1704	6824	7192.88888
18.70528	8517	18.70528	1692	6825	7194.99817
18.73155	8592	18.73155	1687	6905	7280.40434
18.75781	8640	18.75781	1692	6948	7326.81958
18.78407	8524	18.78407	1666	6858	7232.97768
18.81033	8488	18.81033	1656	6832	7206.61869
18.83659	8731	18.83659	1717	7014	7399.69084
18.86285	8631	18.86285	1579	7052	7440.88052
18.88911	8649	18.88911	1668	6981	7367.05588
18.91537	8624	18.91537	1668	6956	7341.7617
18.94163	8519	18.94163	1633	6886	7268.95867
18.96789	8652	18.96789	1634	7018	7409.40102
18.99415	8787	18.99415	1583	7204	7606.90658
19.02041	8832	19.02041	1579	7253	7659.78859
19.04667	8819	19.04667	1549	7270	7678.88793
19.07293	8639	19.07293	1567	7072	7470.86809
19.09919	8821	19.09919	1618	7203	7610.39528
19.12545	8707	19.12545	1651	7056	7456.19815
19.15171	8688	19.15171	1579	7109	7513.33127
19.17797	8672	19.17797	1609	7063	7465.83641
19.20424	8757	19.20424	1564	7193	7604.39468
19.2305	8730	19.2305	1555	7175	7586.50774
19.25676	8789	19.25676	1582	7207	7621.49235
19.28302	8622	19.28302	1615	7007	7411.10891
19.30928	8923	19.30928	1607	7316	7739.0997
19.33554	8689	19.33554	1511	7178	7594.26856
19.3618	8931	19.3618	1577	7354	7781.65478
19.38806	8913	19.38806	1565	7348	7776.4862
19.41432	8959	19.41432	1557	7402	7834.82584
19.44058	8896	19.44058	1653	7243	7667.69527
19.46684	8679	19.46684	1561	7118	7536.51421
19.4931	8732	19.4931	1464	7268	7696.50793
19.51936	8896	19.51936	1476	7420	7858.67009
19.54562	8886	19.54562	1456	7430	7870.46515
19.57188	9038	19.57188	1528	7510	7956.42628
19.59814	8920	19.59814	1499	7421	7863.34159
19.6244	8886	19.6244	1556	7330	7768.11015
19.65066	8841	19.65066	1508	7333	7772.48443
19.67693	8792	19.67693	1518	7274	7711.13547
19.70319	8771	19.70319	1511	7260	7697.48058
19.72945	8937	19.72945	1518	7419	7867.27593
19.75571	8873	19.75571	1566	7307	7749.70613
19.78197	8877	19.78197	1495	7382	7830.46168
19.80823	8845	19.80823	1489	7356	7804.09116
19.83449	8899	19.83449	1526	7373	7823.34022
19.86075	8895	19.86075	1476	7419	7873.3727
19.88701	8934	19.88701	1501	7433	7889.45697
19.91327	8981	19.91327	1516	7465	7924.65596
19.93953	9045	19.93953	1508	7537	8002.33688
19.96579	8849	19.96579	1405	7444	7904.82891
19.99205	8919	19.99205	1459	7460	7923.05768

20.01831	8854	20.01831	1509	7345	7802.14029
20.04457	8912	20.04457	1478	7434	7897.91693
20.07083	8927	20.07083	1449	7478	7945.90924
20.09709	8827	20.09709	1474	7353	7814.3152
20.12336	8832	20.12336	1558	7274	7731.57476
20.14962	8798	20.14962	1420	7378	7843.35194
20.17588	9131	20.17588	1454	7677	8162.49759
20.20214	8889	20.20214	1382	7507	7983.00681
20.2284	8796	20.2284	1408	7388	7857.70308
20.25466	8873	20.25466	1455	7418	7890.85902
20.28092	8823	20.28092	1433	7390	7862.31982
20.30718	8801	20.30718	1501	7300	7767.79982
20.33344	8820	20.33344	1444	7376	7849.91682
20.3597	8823	20.3597	1426	7397	7873.51813
20.38596	8780	20.38596	1446	7334	7807.70273
20.41222	8906	20.41222	1392	7514	8000.60429
20.43848	8991	20.43848	1354	7637	8132.86776
20.46474	8889	20.46474	1355	7534	8024.46229
20.491	8789	20.491	1362	7427	7911.76243
20.51726	8846	20.51726	1399	7447	7934.33881
20.54352	8915	20.54352	1410	7505	7997.41702
20.56978	8837	20.56978	1432	7405	7892.12311
20.59605	8845	20.59605	1435	7410	7898.72191
20.62231	8832	20.62231	1320	7512	8008.73837
20.64857	8787	20.64857	1446	7341	7827.69233
20.67483	8780	20.67483	1414	7366	7855.6173
20.70109	8573	20.70109	1442	7131	7606.22564
20.72735	8824	20.72735	1433	7391	7884.82792
20.75361	8830	20.75361	1379	7451	7950.12421
20.77987	8723	20.77987	1353	7370	7864.97336
20.80613	8716	20.80613	1379	7337	7831.02822
20.83239	8708	20.83239	1392	7316	7809.88346
20.85865	8572	20.85865	1330	7242	7732.14602
20.88491	8660	20.88491	1345	7315	7811.35925
20.91117	8761	20.91117	1419	7342	7841.47027
20.93743	8596	20.93743	1377	7219	7711.3619
20.96369	8678	20.96369	1382	7296	7794.88793
20.98995	8694	20.98995	1232	7462	7973.54384
21.01621	8631	21.01621	1393	7238	7735.45557
21.04247	8735	21.04247	1343	7392	7901.33609
21.06874	8564	21.06874	1338	7226	7725.16702
21.095	8572	21.095	1333	7239	7740.33799
21.12126	8749	21.12126	1371	7378	7890.26357
21.14752	8617	21.14752	1261	7356	7868.03306
21.17378	8746	21.17378	1420	7326	7837.23826
21.20004	8633	21.20004	1341	7292	7802.15474
21.2263	8468	21.2263	1294	7174	7677.16932
21.25256	8827	21.25256	1322	7505	8032.71531
21.27882	8515	21.27882	1332	7183	7689.34878
21.30508	8476	21.30508	1342	7134	7638.16258
21.33134	8476	21.33134	1351	7125	7629.79458
21.3576	8502	21.3576	1292	7210	7722.10155
21.38386	8477	21.38386	1286	7191	7703.03522
21.41012	8495	21.41012	1308	7187	7700.03456
21.43638	8478	21.43638	1265	7213	7729.18105
21.46264	8650	21.46264	1332	7318	7843.00615

21.4889	8533	21.4889	1316	7217	7736.05488
21.51517	8412	21.51517	1364	7048	7556.1662
21.54143	8487	21.54143	1317	7170	7688.2521
21.56769	8401	21.56769	1300	7101	7615.54362
21.59395	8414	21.59395	1304	7110	7626.47795
21.62021	8297	21.62021	1274	7023	7534.42634
21.64647	8194	21.64647	1258	6936	7442.34495
21.67273	8352	21.67273	1319	7033	7547.69948
21.69899	8285	21.69899	1260	7025	7540.38753
21.72525	8343	21.72525	1242	7101	7623.25223
21.75151	8302	21.75151	1262	7040	7559.04548
21.77777	8141	21.77777	1239	6902	7412.12714
21.80403	8152	21.80403	1253	6899	7410.16263
21.83029	8284	21.83029	1295	6989	7508.10622
21.85655	8349	21.85655	1262	7087	7614.67999
21.88281	8207	21.88281	1257	6950	7468.75078
21.90907	8152	21.90907	1274	6878	7392.6366
21.93533	8121	21.93533	1267	6854	7368.098
21.96159	8254	21.96159	1266	6988	7513.43233
21.98786	8166	21.98786	1222	6944	7467.40092
22.01412	8088	22.01412	1235	6853	7370.8037
22.04038	8118	22.04038	1172	6946	7472.11129
22.06664	7963	22.06664	1248	6715	7224.8543
22.0929	8112	22.0929	1268	6844	7364.91406
22.11916	8037	22.11916	1283	6754	7269.314
22.14542	7965	22.14542	1218	6747	7263.03029
22.17168	7926	22.17168	1228	6698	7211.52551
22.19794	8019	22.19794	1257	6762	7281.68867
22.2242	8135	22.2242	1196	6939	7473.58279
22.25046	7928	22.25046	1242	6686	7202.33703
22.27672	7999	22.27672	1220	6779	7303.78349
22.30298	7994	22.30298	1194	6800	7327.67908
22.32924	7988	22.32924	1180	6808	7337.57292
22.3555	7793	22.3555	1228	6565	7076.89987
22.38176	7981	22.38176	1223	6758	7286.21578
22.40802	7726	22.40802	1213	6513	7023.28874
22.43428	7693	22.43428	1244	6449	6955.48645
22.46055	7807	22.46055	1239	6568	7085.06837
22.48681	8021	22.48681	1242	6779	7313.95675
22.51307	7849	22.51307	1216	6633	7157.68673
22.53933	7761	22.53933	1212	6549	7068.27923
22.56559	7889	22.56559	1144	6745	7281.09609
22.59185	7859	22.59185	1232	6627	7154.97241
22.61811	7614	22.61811	1192	6422	6934.85781
22.64437	7597	22.64437	1222	6375	6885.31475
22.67063	7475	22.67063	1153	6322	6829.27393
22.69689	7688	22.69689	1157	6531	7056.28706
22.72315	7637	22.72315	1207	6430	6948.38905
22.74941	7527	22.74941	1130	6397	6913.94922
22.77567	7618	22.77567	1122	6496	7022.19062
22.80193	7637	22.80193	1184	6453	6976.94194
22.82819	7602	22.82819	1202	6400	6920.8645
22.85445	7546	22.85445	1183	6363	6882.0735
22.88071	7316	22.88071	1169	6147	6649.6332
22.90698	7519	22.90698	1196	6323	6841.24013
22.93324	7329	22.93324	1162	6167	6673.6413

22.9595	7495	22.9595	1125	6370	6894.54618
22.98576	7377	22.98576	1166	6211	6723.65167
23.01202	7366	23.01202	1170	6196	6708.61072
23.03828	7308	23.03828	1185	6123	6630.75575
23.06454	7411	23.06454	1191	6220	6737.00433
23.0908	7392	23.0908	1141	6251	6771.79331
23.11706	7293	23.11706	1171	6122	6633.23458
23.14332	7238	23.14332	1169	6069	6576.98856
23.16958	7239	23.16958	1219	6020	6525.05897
23.19584	7259	23.19584	1076	6183	6702.93918
23.2221	7256	23.2221	1158	6098	6611.98131
23.24836	7144	23.24836	1159	5985	6490.62621
23.27462	7195	23.27462	1097	6098	6614.36556
23.30088	7171	23.30088	1116	6055	6568.91032
23.32714	6938	23.32714	1079	5859	6357.424
23.3534	6962	23.3534	1090	5872	6372.6828
23.37967	6878	23.37967	1184	5694	6180.62474
23.40593	6988	23.40593	1158	5830	6329.39512
23.43219	7124	23.43219	1120	6004	6519.48303
23.45845	7049	23.45845	1081	5968	6481.56968
23.48471	6976	23.48471	1135	5841	6344.79467
23.51097	6905	23.51097	1140	5765	6263.37978
23.53723	6723	23.53723	1085	5638	6126.51719
23.56349	6795	23.56349	1081	5714	6210.23526
23.58975	6922	23.58975	1077	5845	6353.7723
23.61601	6950	23.61601	1102	5848	6358.1957
23.64227	6747	23.64227	1060	5687	6184.28127
23.66853	6788	23.66853	1100	5688	6186.50193
23.69479	6716	23.69479	1124	5592	6083.20384
23.72105	6758	23.72105	1084	5674	6173.53993
23.74731	6692	23.74731	1154	5538	6026.67382
23.77357	6726	23.77357	1124	5602	6097.44269
23.79983	6749	23.79983	1141	5608	6105.09739
23.82609	6675	23.82609	1140	5535	6026.73738
23.85236	6515	23.85236	1105	5410	5891.71919
23.87862	6731	23.87862	1018	5713	6222.84827
23.90488	6642	23.90488	1084	5558	6055.13494
23.93114	6592	23.93114	1107	5485	5976.7115
23.9574	6593	23.9574	1081	5512	6007.24477
23.98366	6653	23.98366	1073	5580	6082.48236
24.00992	6545	24.00992	1087	5458	5950.60075
24.03618	6488	24.03618	1091	5397	5885.18884
24.06244	6434	24.06244	1025	5409	5899.37157
24.0887	6354	24.0887	1122	5232	5707.38764
24.11496	6292	24.11496	1072	5220	5695.35876
24.14122	6428	24.14122	1003	5425	5920.13151
24.16748	6405	24.16748	1034	5371	5862.29778
24.19374	6249	24.19374	1061	5188	5663.61707
24.22	6472	24.22	1053	5419	5916.90149
24.24626	6275	24.24626	1003	5272	5757.47345
24.27252	6401	24.27252	1101	5300	5789.13727
24.29878	6189	24.29878	1094	5095	5566.2625
24.32505	6242	24.32505	1093	5149	5626.31424
24.35131	6239	24.35131	1048	5191	5673.27455
24.37757	6106	24.37757	1081	5025	5492.88617
24.40383	6113	24.40383	1026	5087	5561.70709

24.43009	6248	24.43009	1030	5218	5706.00795
24.45635	6204	24.45635	1021	5183	5668.8049
24.48261	6208	24.48261	1080	5128	5609.70991
24.50887	6157	24.50887	996	5161	5646.87809
24.53513	5935	24.53513	1049	4886	5347.00097
24.56139	6269	24.56139	1022	5247	5743.15044
24.58765	6270	24.58765	973	5297	5798.97866
24.61391	6109	24.61391	1061	5048	5527.43156
24.64017	5983	24.64017	1033	4950	5421.15466
24.66643	6003	24.66643	954	5049	5530.63021
24.69269	5981	24.69269	1051	4930	5401.3075
24.71895	6031	24.71895	1088	4943	5416.58306
24.74521	6075	24.74521	1024	5051	5535.98698
24.77148	5994	24.77148	957	5037	5521.69759
24.79774	5978	24.79774	1000	4978	5458.06389
24.824	5991	24.824	1025	4966	5445.94906
24.85026	6015	24.85026	1014	5001	5485.38268
24.87652	5986	24.87652	1029	4957	5438.16391
24.90278	5900	24.90278	963	4937	5417.26251
24.92904	5907	24.92904	1035	4872	5346.96686
24.9553	5928	24.9553	1008	4920	5400.6851
24.98156	5975	24.98156	925	5050	5544.45358
25.00782	5768	25.00782	1032	4736	5200.71157
25.03408	5957	25.03408	1015	4942	5427.97198
25.06034	5882	25.06034	993	4889	5370.79722
25.0866	5936	25.0866	1018	4918	5403.69945
25.11286	5777	25.11286	995	4782	5255.28479
25.13912	5842	25.13912	969	4873	5356.32844
25.16538	5758	25.16538	981	4777	5251.8246
25.19164	5769	25.19164	1039	4730	5201.16195
25.2179	5809	25.2179	996	4813	5293.45764
25.24417	5961	25.24417	972	4989	5488.09359
25.27043	5912	25.27043	988	4924	5417.64514
25.29669	5867	25.29669	996	4871	5360.37565
25.32295	5792	25.32295	988	4804	5287.67508
25.34921	5767	25.34921	975	4792	5275.49623
25.37547	5782	25.37547	984	4798	5283.1334
25.40173	5775	25.40173	1024	4751	5232.40401
25.42799	5793	25.42799	1007	4786	5271.98201
25.45425	5871	25.45425	919	4952	5455.90661
25.48051	5636	25.48051	972	4664	5139.60781
25.50677	5825	25.50677	953	4872	5369.87213
25.53303	5738	25.53303	1002	4736	5220.99966
25.55929	5727	25.55929	987	4740	5226.43676
25.58555	5723	25.58555	917	4806	5300.25288
25.61181	5489	25.61181	994	4495	4958.24596
25.63807	5640	25.63807	934	4706	5192.01477
25.66433	5491	25.66433	929	4562	5034.13646
25.69059	5454	25.69059	950	4504	4971.11569
25.71686	5568	25.71686	988	4580	5055.99729
25.74312	5516	25.74312	971	4545	5018.35277
25.76938	5519	25.76938	963	4556	5031.49494
25.79564	5429	25.79564	1011	4418	4880.05977
25.8219	5404	25.8219	922	4482	4951.73582
25.84816	5592	25.84816	964	4628	5114.05305
25.87442	5493	25.87442	921	4572	5053.17623

25.90068	5510	25.90068	909	4601	5086.24036
25.92694	5489	25.92694	946	4543	5023.12385
25.9532	5479	25.9532	966	4513	4990.94823
25.97946	5305	25.97946	910	4395	4861.42146
26.00572	5432	26.00572	963	4469	4944.26216
26.03198	5452	26.03198	989	4463	4938.61128
26.05824	5497	26.05824	929	4568	5055.81242
26.0845	5418	26.0845	912	4506	4988.19043
26.11076	5349	26.11076	885	4464	4942.6867
26.13702	5533	26.13702	911	4622	5118.65639
26.16329	5268	26.16329	918	4350	4818.39623
26.18955	5312	26.18955	943	4369	4840.41497
26.21581	5429	26.21581	950	4479	4963.28244
26.24207	5346	26.24207	920	4426	4905.53968
26.26833	5355	26.26833	937	4418	4897.65997
26.29459	5228	26.29459	932	4296	4763.37536
26.32085	5392	26.32085	909	4483	4971.72347
26.34711	5247	26.34711	861	4386	4865.13198
26.37337	5214	26.37337	881	4333	4807.31455
26.39963	5209	26.39963	959	4250	4716.18368
26.42589	5348	26.42589	893	4455	4944.67212
26.45215	5342	26.45215	926	4416	4902.37968
26.47841	5390	26.47841	912	4478	4972.21771
26.50467	5265	26.50467	892	4373	4856.61605
26.53093	5290	26.53093	849	4441	4933.13945
26.55719	5324	26.55719	944	4380	4866.37008
26.58345	5140	26.58345	955	4185	4650.66405
26.60971	5313	26.60971	937	4376	4863.90834
26.63598	5436	26.63598	934	4502	5004.97831
26.66224	5302	26.66224	943	4359	4846.99194
26.6885	5293	26.6885	875	4418	4913.60158
26.71476	5066	26.71476	957	4109	4570.87396
26.74102	5222	26.74102	861	4361	4852.19389
26.76728	5227	26.76728	884	4343	4833.15723
26.79354	5155	26.79354	861	4294	4779.60765
26.8198	5224	26.8198	859	4365	4859.63495
26.84606	5110	26.84606	875	4235	4715.87281
26.87232	5204	26.87232	893	4311	4801.49013
26.89858	5158	26.89858	841	4317	4809.16296
26.92484	5205	26.92484	871	4334	4829.0962
26.9511	5194	26.9511	856	4338	4834.55029
26.97736	5025	26.97736	922	4103	4573.5951
27.00362	5135	27.00362	879	4256	4745.1239
27.02988	5064	27.02988	868	4196	4679.19599
27.05614	4954	27.05614	903	4051	4518.43352
27.0824	5105	27.0824	878	4227	4715.71855
27.10867	4960	27.10867	885	4075	4547.08734
27.13493	5176	27.13493	866	4310	4810.31022
27.16119	4972	27.16119	800	4172	4657.2583
27.18745	5013	27.18745	855	4158	4642.595
27.21371	4988	27.21371	809	4179	4667.01344
27.23997	4990	27.23997	822	4168	4655.69837
27.26623	5129	27.26623	881	4248	4746.04833
27.29249	5000	27.29249	867	4133	4618.52878
27.31875	4954	27.31875	863	4091	4572.54941
27.34501	5035	27.34501	866	4169	4660.70462

27.37127	4905	27.37127	800	4105	4590.11621
27.39753	4886	27.39753	890	3996	4469.17037
27.42379	4935	27.42379	881	4054	4534.98825
27.45005	4974	27.45005	830	4144	4636.6385
27.47631	4971	27.47631	812	4159	4654.39842
27.50257	4961	27.50257	867	4094	4582.61844
27.52883	5145	27.52883	860	4285	4797.42273
27.5551	5048	27.5551	824	4224	4730.12318
27.58136	5016	27.58136	833	4183	4685.19707
27.60762	4893	27.60762	848	4045	4531.58425
27.63388	4935	27.63388	856	4079	4570.63826
27.66014	4924	27.66014	832	4092	4586.17328
27.6864	4838	27.6864	847	3991	4473.9212
27.71266	4933	27.71266	789	4144	4646.41711
27.73892	4830	27.73892	853	3977	4460.11401
27.76518	4937	27.76518	853	4084	4581.08239
27.79144	4800	27.79144	840	3960	4442.93166
27.8177	4884	27.8177	871	4013	4503.35062
27.84396	4875	27.84396	775	4100	4601.95842
27.87022	4823	27.87022	780	4043	4538.94462
27.89648	4808	27.89648	877	3931	4414.14478
27.92274	4967	27.92274	864	4103	4608.26569
27.949	4937	27.949	836	4101	4607.00097
27.97526	4840	27.97526	805	4035	4533.82436
28.00152	4844	28.00152	887	3957	4447.13075
28.02779	4698	28.02779	759	3939	4427.84696
28.05405	4770	28.05405	759	4011	4509.74653
28.08031	4825	28.08031	858	3967	4461.22984
28.10657	4850	28.10657	776	4074	4582.54171
28.13283	4796	28.13283	810	3986	4484.51805
28.15909	4804	28.15909	816	3988	4487.73072
28.18535	4677	28.18535	829	3848	4331.11722
28.21161	4743	28.21161	781	3962	4460.3882
28.23787	4744	28.23787	822	3922	4416.30607
28.26413	4645	28.26413	804	3841	4326.02821
28.29039	4763	28.29039	794	3969	4471.15455
28.31665	4858	28.31665	814	4044	4556.62563
28.34291	4601	28.34291	847	3754	4230.7773
28.36917	4902	28.36917	850	4052	4567.61096
28.39543	4730	28.39543	782	3948	4451.33892
28.42169	4734	28.42169	812	3922	4422.98059
28.44795	4622	28.44795	781	3841	4332.57165
28.47421	4756	28.47421	827	3929	4432.79405
28.50048	4730	28.50048	789	3941	4447.29682
28.52674	4634	28.52674	835	3799	4287.98449
28.553	4671	28.553	795	3876	4375.8456
28.57926	4779	28.57926	780	3999	4515.68885
28.60552	4603	28.60552	887	3716	4197.03675
28.63178	4649	28.63178	767	3882	4385.48001
28.65804	4474	28.65804	781	3693	4172.87645
28.6843	4625	28.6843	739	3886	4391.91276
28.71056	4597	28.71056	771	3826	4325.0451
28.73682	4557	28.73682	824	3733	4220.83629
28.76308	4546	28.76308	742	3804	4302.05486
28.78934	4598	28.78934	750	3848	4352.76774
28.8156	4603	28.8156	761	3842	4346.93216

28.84186	4660	28.84186	818	3842	4347.88461
28.86812	4545	28.86812	706	3839	4345.44227
28.89438	4531	28.89438	754	3777	4276.20146
28.92064	4550	28.92064	836	3714	4205.79836
28.94691	4512	28.94691	803	3709	4201.0595
28.97317	4466	28.97317	764	3702	4194.05325
28.99943	4527	28.99943	746	3781	4284.49664
29.02569	4517	29.02569	772	3745	4244.63773
29.05195	4489	29.05195	768	3721	4218.36575
29.07821	4423	29.07821	754	3669	4160.33311
29.10447	4614	29.10447	784	3830	4343.85253
29.13073	4457	29.13073	755	3702	4199.60743
29.15699	4439	29.15699	711	3728	4230.03774
29.18325	4631	29.18325	746	3885	4409.15631
29.20951	4467	29.20951	780	3687	4185.36963
29.23577	4605	29.23577	796	3809	4324.81901
29.26203	4463	29.26203	807	3656	4152.02072
29.28829	4488	29.28829	738	3750	4259.71976
29.31455	4335	29.31455	736	3599	4089.10364
29.34081	4524	29.34081	787	3737	4246.84055
29.36707	4466	29.36707	786	3680	4182.99493
29.39333	4521	29.39333	721	3800	4320.35917
29.4196	4443	29.4196	698	3745	4258.77691
29.44586	4353	29.44586	706	3647	4148.25762
29.47212	4375	29.47212	743	3632	4132.11842
29.49838	4316	29.49838	761	3555	4045.41949
29.52464	4450	29.52464	714	3736	4252.33957
29.5509	4312	29.5509	695	3617	4117.8144
29.57716	4297	29.57716	738	3559	4052.69117
29.60342	4377	29.60342	738	3639	4144.71733
29.62968	4297	29.62968	773	3524	4014.636
29.65594	4355	29.65594	780	3575	4073.65091
29.6822	4310	29.6822	808	3502	3991.36522
29.70846	4233	29.70846	798	3435	3915.883
29.73472	4343	29.73472	746	3597	4101.4849
29.76098	4282	29.76098	747	3535	4031.697
29.78724	4256	29.78724	722	3534	4031.46483
29.8135	4241	29.8135	748	3493	3985.59214
29.83976	4279	29.83976	712	3567	4070.94645
29.86602	4225	29.86602	712	3513	4010.22294
29.89229	4235	29.89229	687	3548	4051.09232
29.91855	4294	29.91855	764	3530	4031.4518
29.94481	4287	29.94481	731	3556	4062.06463
29.97107	4281	29.97107	703	3578	4088.12154
29.99733	4169	29.99733	718	3451	3943.90897
30.02359	4266	30.02359	688	3578	4089.97632
30.04985	4216	30.04985	765	3451	3945.69968
30.07611	4293	30.07611	687	3606	4123.85582
30.10237	4287	30.10237	709	3578	4092.76534
30.12863	4213	30.12863	712	3501	4005.59883
30.15489	4245	30.15489	737	3508	4014.5219
30.18115	4306	30.18115	656	3650	4177.97745
30.20741	4237	30.20741	674	3563	4079.3231
30.23367	4259	30.23367	657	3602	4124.91611
30.25993	4302	30.25993	653	3649	4179.69391
30.28619	4163	30.28619	688	3475	3981.29814

30.31245	3969	30.31245	683	3286	3765.6227
30.33871	4227	30.33871	704	3523	4038.13945
30.36498	4164	30.36498	734	3430	3932.44167
30.39124	4178	30.39124	795	3383	3879.44626
30.4175	4193	30.4175	726	3467	3976.68537
30.44376	4214	30.44376	694	3520	4038.4041
30.47002	4153	30.47002	653	3500	4016.38143
30.49628	4165	30.49628	664	3501	4018.45293
30.52254	4065	30.52254	715	3350	3846.01988
30.5488	4128	30.5488	702	3426	3934.17879
30.57506	4127	30.57506	721	3406	3912.11369
30.60132	4200	30.60132	743	3457	3971.60791
30.62758	4144	30.62758	702	3442	3955.28781
30.65384	4125	30.65384	684	3441	3955.0521
30.6801	4152	30.6801	712	3440	3954.81673
30.70636	4046	30.70636	700	3346	3847.63897
30.73262	4202	30.73262	693	3509	4036.01041
30.75888	4262	30.75888	660	3602	4143.93775
30.78514	4028	30.78514	691	3337	3839.95733
30.81141	4086	30.81141	719	3367	3875.37792
30.83767	4025	30.83767	661	3364	3872.82395
30.86393	4066	30.86393	683	3383	3895.60275
30.89019	4019	30.89019	734	3285	3783.63306
30.91645	4009	30.91645	726	3283	3782.20936
30.94271	4027	30.94271	714	3313	3817.65989
30.96897	4047	30.96897	689	3358	3870.41632
30.99523	4044	30.99523	658	3386	3903.59909
31.02149	3946	31.02149	680	3266	3766.13403
31.04775	4021	31.04775	688	3333	3844.29152
31.07401	4067	31.07401	742	3325	3835.96056
31.10027	4041	31.10027	658	3383	3903.78631
31.12653	3994	31.12653	647	3347	3863.14829
31.15279	3990	31.15279	699	3291	3799.40203
31.17905	4039	31.17905	695	3344	3861.49442
31.20531	4067	31.20531	730	3337	3854.31492
31.23157	3943	31.23157	693	3250	3754.70888
31.25783	3934	31.25783	647	3287	3798.34672
31.2841	4057	31.2841	635	3422	3955.27761
31.31036	3975	31.31036	680	3295	3809.38197
31.33662	3996	31.33662	675	3321	3840.34453
31.36288	3889	31.36288	671	3218	3722.1137
31.38914	3997	31.38914	681	3316	3836.36988
31.4154	4042	31.4154	711	3331	3854.63278
31.44166	3988	31.44166	589	3399	3934.2508
31.46792	3990	31.46792	599	3391	3925.91813
31.49418	3980	31.49418	684	3296	3816.83421
31.52044	3903	31.52044	671	3232	3743.60624
31.5467	3888	31.5467	686	3202	3709.73533
31.57296	3982	31.57296	661	3321	3848.51635
31.59922	3918	31.59922	619	3299	3823.92802
31.62548	3910	31.62548	658	3252	3770.34364
31.65174	3823	31.65174	715	3108	3604.2465
31.678	3910	31.678	665	3245	3764.01483
31.70426	3861	31.70426	660	3201	3713.85993
31.73052	3942	31.73052	665	3277	3802.94094
31.75679	3964	31.75679	630	3334	3870.01011

31.78305	3950	31.78305	647	3303	3834.93948
31.80931	3955	31.80931	665	3290	3820.75639
31.83557	3837	31.83557	624	3213	3732.22447
31.86183	3938	31.86183	633	3305	3840.00815
31.88809	3857	31.88809	637	3220	3742.14213
31.91435	3896	31.91435	658	3238	3763.96041
31.94061	3958	31.94061	646	3312	3850.90138
31.96687	3855	31.96687	675	3180	3698.30843
31.99313	3924	31.99313	700	3224	3750.37807
32.01939	3901	32.01939	679	3222	3748.94989
32.04565	3821	32.04565	677	3144	3659.07061
32.07191	3797	32.07191	639	3158	3676.24632
32.09817	3789	32.09817	690	3099	3608.43054
32.12443	3920	32.12443	652	3268	3806.12623
32.15069	3951	32.15069	636	3315	3861.79407
32.17695	3996	32.17695	665	3331	3881.36711
32.20322	3885	32.20322	643	3242	3778.5718
32.22948	3851	32.22948	632	3219	3752.66933
32.25574	3771	32.25574	616	3155	3678.94595
32.282	3852	32.282	616	3236	3774.30812
32.30826	3818	32.30826	584	3234	3772.88631
32.33452	3899	32.33452	582	3317	3870.65186
32.36078	3825	32.36078	668	3157	3684.83655
32.38704	3947	32.38704	644	3303	3856.18
32.4133	3900	32.4133	653	3247	3791.71914
32.43956	3811	32.43956	627	3184	3719.05115
32.46582	3834	32.46582	596	3238	3783.04256
32.49208	3915	32.49208	619	3296	3851.73985
32.51834	3860	32.51834	677	3183	3720.58999
32.5446	3812	32.5446	585	3227	3772.93777
32.57086	3894	32.57086	651	3243	3792.56645
32.59712	3903	32.59712	651	3252	3804.01683
32.62338	3838	32.62338	630	3208	3753.46151
32.64964	3764	32.64964	620	3144	3679.47561
32.67591	3774	32.67591	622	3152	3689.73737
32.70217	3811	32.70217	639	3172	3714.05516
32.72843	3794	32.72843	686	3108	3640.00664
32.75469	3892	32.75469	668	3224	3776.78502
32.78095	3786	32.78095	639	3147	3687.48371
32.80721	3840	32.80721	651	3189	3737.61094
32.83347	3884	32.83347	641	3243	3801.83092
32.85973	3867	32.85973	625	3242	3801.58939
32.88599	3855	32.88599	640	3215	3770.85289
32.91225	3718	32.91225	654	3064	3594.62723
32.93851	3687	32.93851	601	3086	3621.32561
32.96477	3733	32.96477	677	3056	3587.0021
32.99103	3836	32.99103	619	3217	3776.9048
33.01729	3887	33.01729	630	3257	3824.80679
33.04355	3836	33.04355	664	3172	3725.90482
33.06981	3978	33.06981	602	3376	3966.50423
33.09607	3730	33.09607	624	3106	3650.17688
33.12233	3772	33.12233	631	3141	3692.21889
33.1486	3897	33.1486	655	3242	3811.88358
33.17486	3782	33.17486	590	3192	3754.02088
33.20112	3874	33.20112	519	3355	3946.69513
33.22738	3778	33.22738	575	3203	3768.81931

33.25364	3914	33.25364	570	3344	3935.70047
33.2799	3734	33.2799	613	3121	3674.15099
33.30616	3888	33.30616	608	3280	3862.28757
33.33242	3795	33.33242	632	3163	3725.43984
33.35868	3796	33.35868	607	3189	3756.99443
33.38494	3698	33.38494	616	3082	3631.83747
33.4112	3750	33.4112	579	3171	3737.64303
33.43746	3849	33.43746	554	3295	3884.7661
33.46372	3813	33.46372	649	3164	3731.24595
33.48998	3851	33.48998	628	3223	3801.76898
33.51624	3837	33.51624	607	3230	3810.97433
33.5425	3831	33.5425	597	3234	3816.64415
33.56876	3964	33.56876	607	3357	3962.79144
33.59503	3705	33.59503	589	3116	3679.21879
33.62129	3877	33.62129	661	3216	3798.24142
33.64755	3855	33.64755	598	3257	3847.62477
33.67381	3703	33.67381	632	3071	3628.80195
33.70007	3865	33.70007	600	3265	3859.00376
33.72633	3800	33.72633	583	3217	3803.2223
33.75259	3779	33.75259	584	3195	3778.15886
33.77885	3785	33.77885	594	3191	3774.37396
33.80511	3845	33.80511	629	3216	3804.89786
33.83137	3984	33.83137	552	3432	4061.46907
33.85763	3912	33.85763	569	3343	3957.13825
33.88389	3832	33.88389	612	3220	3812.49922
33.91015	3883	33.91015	584	3299	3907.01717
33.93641	3886	33.93641	583	3303	3912.73792
33.96267	3836	33.96267	591	3245	3844.99816
33.98893	3890	33.98893	572	3318	3932.48554
34.01519	3932	34.01519	564	3368	3992.75094
34.04145	3852	34.04145	608	3244	3846.71877
34.06772	3884	34.06772	573	3311	3927.15727
34.09398	3816	34.09398	576	3240	3843.91444
34.12024	3815	34.12024	577	3238	3842.51175
34.1465	3843	34.1465	547	3296	3912.32827
34.17276	3863	34.17276	533	3330	3953.68542
34.19902	3919	34.19902	602	3317	3939.24697
34.22528	3977	34.22528	551	3426	4069.72464
34.25154	3938	34.25154	577	3361	3993.52287
34.2778	3918	34.2778	536	3382	4019.49349
34.30406	3903	34.30406	589	3314	3939.67469
34.33032	3862	34.33032	561	3301	3925.21618
34.35658	3898	34.35658	617	3281	3902.42489
34.38284	3884	34.38284	569	3315	3943.86635
34.4091	3819	34.4091	590	3229	3842.52854
34.43536	3766	34.43536	602	3164	3766.13601
34.46162	4043	34.46162	565	3478	4140.94668
34.48788	3852	34.48788	601	3251	3871.66376
34.51414	3937	34.51414	611	3326	3961.99185
34.54041	3901	34.54041	598	3303	3935.59721
34.56667	3909	34.56667	567	3342	3983.08267
34.59293	3977	34.59293	575	3402	4055.62746
34.61919	4019	34.61919	612	3407	4062.62575
34.64545	3770	34.64545	581	3189	3803.64702
34.67171	3907	34.67171	548	3359	4007.43756
34.69797	3864	34.69797	600	3264	3895.09495

34.72423	3942	34.72423	568	3374	4027.39459
34.75049	3988	34.75049	561	3427	4091.70656
34.77675	3867	34.77675	564	3303	3944.66647
34.80301	3873	34.80301	552	3321	3967.18083
34.82927	3967	34.82927	561	3406	4069.7641
34.85553	3940	34.85553	568	3372	4030.17307
34.88179	3923	34.88179	564	3359	4015.66745
34.90805	4020	34.90805	586	3434	4106.38532
34.93431	3973	34.93431	606	3367	4027.30259
34.96057	3887	34.96057	524	3363	4023.55384
34.98683	3874	34.98683	588	3286	3932.44248
35.0131	3992	35.0131	584	3408	4079.49441
35.03936	3834	35.03936	574	3260	3903.3398
35.06562	3962	35.06562	541	3421	4097.16923
35.09188	4026	35.09188	555	3471	4158.12535
35.11814	3936	35.11814	557	3379	4048.95878
35.1444	3914	35.1444	578	3336	3998.4666
35.17066	3866	35.17066	543	3323	3983.91538
35.19692	3836	35.19692	548	3288	3942.97453
35.22318	3923	35.22318	561	3362	4032.75963
35.24944	3955	35.24944	564	3391	4068.59956
35.2757	3769	35.2757	553	3216	3859.63107
35.30196	3939	35.30196	574	3365	4039.49881
35.32822	3838	35.32822	560	3278	3936.08159
35.35448	3896	35.35448	543	3353	4027.18415
35.38074	3974	35.38074	523	3451	4145.96616
35.407	3760	35.407	547	3213	3861.04127
35.43326	3993	35.43326	551	3442	4137.30534
35.45953	3881	35.45953	565	3316	3986.89028
35.48579	3883	35.48579	548	3335	4010.77889
35.51205	3825	35.51205	563	3262	3924.00926
35.53831	3865	35.53831	551	3314	3987.60215
35.56457	3936	35.56457	584	3352	4034.37852
35.59083	3891	35.59083	577	3314	3989.68417
35.61709	3857	35.61709	601	3256	3920.88275
35.64335	3914	35.64335	557	3357	4043.56391
35.66961	3759	35.66961	569	3190	3843.41454
35.69587	3937	35.69587	511	3426	4128.83516
35.72213	4023	35.72213	545	3478	4192.60038
35.74839	3989	35.74839	534	3455	4165.96592
35.77465	3852	35.77465	548	3304	3984.93769
35.80091	4046	35.80091	510	3536	4265.87035
35.82717	3876	35.82717	560	3316	4001.50977
35.85343	3909	35.85343	542	3367	4064.11984
35.87969	3838	35.87969	529	3309	3995.16067
35.90595	3790	35.90595	530	3260	3937.03468
35.93222	3932	35.93222	548	3384	4087.86191
35.95848	3909	35.95848	583	3326	4018.85552
35.98474	3855	35.98474	598	3257	3936.51815
36.011	3833	36.011	514	3319	4012.51036
36.03726	3904	36.03726	559	3345	4045.00922
36.06352	3976	36.06352	566	3410	4124.69952
36.08978	3822	36.08978	479	3343	4044.72427
36.11604	3855	36.11604	534	3321	4019.16737
36.1423	3930	36.1423	563	3367	4075.91453
36.16856	3879	36.16856	593	3286	3978.91178

36.19482	3785	36.19482	496	3289	3983.59784
36.22108	3847	36.22108	482	3365	4076.72682
36.24734	3920	36.24734	545	3375	4089.92468
36.2736	3879	36.2736	572	3307	4008.5821
36.29986	3898	36.29986	575	3323	4029.04434
36.32612	3879	36.32612	562	3317	4022.83629
36.35238	3770	36.35238	541	3229	3917.14978
36.37864	3875	36.37864	544	3331	4041.96059
36.40491	3855	36.40491	549	3306	4012.6905
36.43117	3956	36.43117	568	3388	4113.312
36.45743	3906	36.45743	531	3375	4098.61879
36.48369	3672	36.48369	528	3144	3819.10721
36.50995	3863	36.50995	509	3354	4075.2851
36.53621	3852	36.53621	505	3347	4067.8632
36.56247	3951	36.56247	544	3407	4141.88954
36.58873	3934	36.58873	529	3405	4140.56217
36.61499	3879	36.61499	510	3369	4097.87855
36.64125	3896	36.64125	546	3350	4075.85589
36.66751	3832	36.66751	505	3327	4048.95377
36.69377	3867	36.69377	523	3344	4070.73051
36.72003	3780	36.72003	533	3247	3953.70719
36.74629	3729	36.74629	510	3219	3920.66181
36.77255	3814	36.77255	457	3357	4089.83702
36.79881	3832	36.79881	472	3360	4094.58844
36.82507	3916	36.82507	525	3391	4133.47341
36.85134	3833	36.85134	540	3293	4015.09229
36.8776	3905	36.8776	494	3411	4160.08329
36.90386	3901	36.90386	532	3369	4109.96274
36.93012	3936	36.93012	563	3373	4115.94767
36.95638	3889	36.95638	523	3366	4108.50961
36.98264	3713	36.98264	548	3165	3864.20951
37.0089	3850	37.0089	508	3342	4081.40989
37.03516	3757	37.03516	473	3284	4011.657
37.06142	3776	37.06142	529	3247	3967.52685
37.08768	3786	37.08768	496	3290	4021.15197
37.11394	3672	37.11394	535	3137	3835.18371
37.1402	3767	37.1402	528	3239	3960.95335
37.16646	3840	37.16646	568	3272	4002.3887
37.19272	3854	37.19272	548	3306	4045.07024
37.21898	3791	37.21898	524	3267	3998.43154
37.24524	3918	37.24524	541	3377	4134.17603
37.2715	3836	37.2715	504	3332	4080.18948
37.29776	3742	37.29776	514	3228	3953.9062
37.32403	3806	37.32403	562	3244	3974.57997
37.35029	3787	37.35029	547	3240	3970.75438
37.37655	3854	37.37655	530	3324	4074.80387
37.40281	3834	37.40281	523	3311	4059.96806
37.42907	3899	37.42907	515	3384	4150.60681
37.45533	3884	37.45533	504	3380	4146.82593
37.48159	3872	37.48159	510	3362	4125.8624
37.50785	3845	37.50785	495	3350	4112.25299
37.53411	3871	37.53411	526	3345	4107.23156
37.56037	3827	37.56037	496	3331	4091.15381
37.58663	3778	37.58663	551	3227	3964.49898
37.61289	3804	37.61289	533	3271	4019.64889
37.63915	3828	37.63915	520	3308	4066.22464

37.66541	3745	37.66541	519	3226	3966.51026
37.69167	3802	37.69167	486	3316	4078.28108
37.71793	3833	37.71793	457	3376	4153.20668
37.74419	3741	37.74419	499	3242	3989.44655
37.77045	3773	37.77045	454	3319	4085.31442
37.79672	3872	37.79672	485	3387	4170.15393
37.82298	3807	37.82298	552	3255	4008.72808
37.84924	3899	37.84924	511	3388	4173.66687
37.8755	3767	37.8755	507	3260	4017.08311
37.90176	3856	37.90176	457	3399	4189.51045
37.92802	3764	37.92802	436	3328	4103.12159
37.95428	3857	37.95428	470	3387	4177.00776
37.98054	3850	37.98054	509	3341	4121.40827
38.0068	3801	38.0068	496	3305	4078.11776
38.03306	3864	38.03306	467	3397	4192.78933
38.05932	3760	38.05932	512	3248	4009.98522
38.08558	3775	38.08558	486	3289	4061.71962
38.11184	3793	38.11184	500	3293	4067.77735
38.1381	3666	38.1381	523	3143	3883.5532
38.16436	3714	38.16436	483	3231	3993.38637
38.19062	3826	38.19062	466	3360	4153.96856
38.21688	3661	38.21688	496	3165	3913.96787
38.24315	3788	38.24315	488	3300	4082.03905
38.26941	3712	38.26941	479	3233	4000.26399
38.29567	3783	38.29567	468	3315	4102.85598
38.32193	3804	38.32193	515	3289	4071.80025
38.34819	3776	38.34819	507	3269	4048.1577
38.37445	3771	38.37445	431	3340	4137.22308
38.40071	3686	38.40071	489	3197	3961.1851
38.42697	3775	38.42697	455	3320	4114.72364
38.45323	3703	38.45323	532	3171	3931.14422
38.47949	3857	38.47949	470	3387	4200.08556
38.50575	3766	38.50575	437	3329	4129.30536
38.53201	3683	38.53201	452	3231	4008.85627
38.55827	3721	38.55827	468	3253	4037.27166
38.58453	3884	38.58453	443	3441	4271.78142
38.61079	3665	38.61079	466	3199	3972.45594
38.63705	3656	38.63705	484	3172	3940.02144
38.66331	3713	38.66331	470	3243	4029.33124
38.68957	3666	38.68957	450	3216	3996.89498
38.71584	3805	38.71584	472	3333	4143.45611
38.7421	3693	38.7421	446	3247	4037.6671
38.76836	3794	38.76836	458	3336	4149.49372
38.79462	3832	38.79462	464	3368	4190.46347
38.82088	3713	38.82088	439	3274	4074.6435
38.84714	3705	38.84714	479	3226	4016.02427
38.8734	3748	38.8734	441	3307	4118.00853
38.89966	3705	38.89966	530	3175	3954.73981
38.92592	3690	38.92592	490	3200	3986.99197
38.95218	3615	38.95218	455	3160	3938.25398
38.97844	3672	38.97844	453	3219	4012.90544
39.0047	3666	39.0047	441	3225	4021.50894
39.03096	3686	39.03096	483	3203	3995.19228
39.05722	3743	39.05722	487	3256	4062.43691
39.08348	3677	39.08348	457	3220	4018.64504
39.10974	3758	39.10974	480	3278	4092.17619

39.136	3697	39.136	468	3229	4032.13512
39.16226	3752	39.16226	487	3265	4078.23204
39.18853	3749	39.18853	464	3285	4104.36422
39.21479	3718	39.21479	492	3226	4031.77893
39.24105	3695	39.24105	483	3212	4015.40884
39.26731	3695	39.26731	474	3221	4027.79076
39.29357	3594	39.29357	482	3112	3892.58195
39.31983	3752	39.31983	502	3250	4066.33911
39.34609	3740	39.34609	470	3270	4092.5133
39.37235	3680	39.37235	460	3220	4031.07045
39.39861	3753	39.39861	544	3209	4018.43049
39.42487	3722	39.42487	505	3217	4029.58284
39.45113	3731	39.45113	480	3251	4073.31819
39.47739	3649	39.47739	457	3192	4000.52185
39.50365	3659	39.50365	417	3242	4064.33248
39.52991	3651	39.52991	450	3201	4014.065
39.55617	3583	39.55617	408	3175	3982.5847
39.58243	3687	39.58243	434	3253	4081.57684
39.60869	3603	39.60869	450	3153	3957.22334
39.63495	3726	39.63495	475	3251	4081.37308
39.66122	3565	39.66122	500	3065	3848.95288
39.68748	3633	39.68748	486	3147	3953.04451
39.71374	3571	39.71374	485	3086	3877.51767
39.74	3710	39.74	477	3233	4063.37146
39.76626	3692	39.76626	429	3263	4102.23853
39.79252	3555	39.79252	450	3105	3904.70752
39.81878	3641	39.81878	458	3183	4003.93177
39.84504	3689	39.84504	460	3229	4062.94791
39.8713	3656	39.8713	431	3225	4059.06648
39.89756	3602	39.89756	428	3174	3996.01086
39.92382	3545	39.92382	455	3090	3891.36137
39.95008	3535	39.95008	478	3057	3850.89719
39.97634	3458	39.97634	507	2951	3718.42608
40.0026	3621	40.0026	497	3124	3937.53549
